# Activation of human spinal locomotor circuitry using transvertebral magnetic stimulation

**DOI:** 10.3389/fnhum.2022.1016064

**Published:** 2022-09-23

**Authors:** Kazutake Kawai, Toshiki Tazoe, Toshimasa Yanai, Kazuyuki Kanosue, Yukio Nishimura

**Affiliations:** ^1^College of Sports Sciences, Nihon University, Tokyo, Japan; ^2^Neural Prosthetics Project, Department of Brain and Neuroscience, Tokyo Metropolitan Institute of Medical Science, Tokyo, Japan; ^3^Faculty of Sport Sciences, Waseda University, Saitama, Japan; ^4^Institute of Health and Sports Science and Medicine, Juntendo University, Chiba, Japan

**Keywords:** spinal cord, locomotion, central pattern generator, magnetic stimulation, human

## Abstract

Transvertebral magnetic stimulation (TVMS) of the human lumbar spinal cord can evoke bilateral rhythmic leg movements, as in walking, supposedly through the activation of spinal locomotor neural circuitry. However, an appropriate stimulus intensity that can effectively drive the human spinal locomotor circuitry to evoke walking-like movements has not been determined. To address this issue, TVMS was delivered over an intervertebral space of the lumbar cord (L1–L3) at different stimulus intensities (10–70% of maximum stimulator output) in healthy human adults. In a stimulus intensity-dependent manner, TVMS evoked two major patterns of rhythmic leg movements in which the left-right movement cycles were coordinated with different phase relationships: hopping-like movements, in which both legs moved in the same direction in phase, and walking-like movements, in which both legs moved alternatively in anti-phase; uncategorized movements were also observed which could not be categorized as either movement type. Even at the same stimulation site, the stimulus-evoked rhythmic movements changed from hopping-like movements to walking-like movements as stimulus intensity was increased. Different leg muscle activation patterns were engaged in the induction of the hopping- and walking-like movements. The magnitude of the evoked hopping- and walking-like movements was positively correlated with stimulus intensity. The human spinal neural circuitry required a higher intensity of magnetic stimulation to produce walking-like leg movements than to produce hopping-like movements. These results suggest that TVMS activates distinct neural modules in the human spinal cord to generate hopping- and walking-like movements.

## Introduction

Today, 200–500 per million people worldwide require rehabilitation due to spinal cord injury (SCI), and 10–80 per million new cases occur each year (Wyndaele and Wyndaele, 2006; Kang et al., 2018). Damaged spinal nerves cannot be completely treated with current medical approaches, but medical technologies are being developed that can profoundly support the daily life of individuals with SCI. Locomotor function can reportedly be reconstructed by applying epidural electrical stimulation to the uninjured part of the lumbosacral spinal cord in humans with SCI (Harkema et al., 2011; Angeli et al., 2018; Gill et al., 2018; Wagner et al., 2018; Rowald et al., 2022), demonstrating that this is an innovative approach for the treatment of patients with chronic severe SCI who had been regarded previously as incurable. However, epidural electrical stimulation requires the invasive implantation of stimulation electrodes. Therefore, it is unlikely to be the first-line treatment for mild gait disorders, and an alternative method is needed to accomplish non-invasive stimulation of the human spinal locomotor circuitry.

Previous studies involving healthy adults have shown that walking-like movements can be induced by transvertebral magnetic stimulation (TVMS) of the lumbar spinal cord (Gerasimenko et al., 2010; Sasada et al., 2014). We also developed a closed-loop TVMS protocol to generate a stimulation pattern similar to the rhythm of walking, thereby improving our ability to induce walking-like movements (Sasada et al., 2014). As tonic TVMS at a fixed frequency has only small population of responders showing the stimulus-evoked walking-like movement (Gerasimenko et al., 2010; Sasada et al., 2014), the rhythmically controlled TVMS is advantageous for inducing gait behavior. The fact that trains of TVMS given to a single spot over the lumbar vertebrae result in alternating rhythmic activation of the intralimb flexor-extensor muscles and left-right homonymous muscles suggests that these walking-like movements are produced by the activation of the premotoneuronal spinal network. However, a systematic investigation remains to be conducted to determine an appropriate intensity of TVMS to drive the human spinal neural circuitry efficiently for evoking walking-like movements.

Thus, in the present study, we systematically investigated the effect of TVMS intensity on stimulus-evoked leg movements in healthy humans. We demonstrated that the intensity of TVMS delivered over the lumbar spinal cord was a critical factor determining the phase relationship of artificially evoked bilateral rhythmic leg movements. Our results could contribute to the development of an innovative neurorehabilitation method via the application of non-invasive TVMS to the lumbar spinal cord for impaired walking after SCI or cerebral infarction.

## Materials and methods

### Participants

Seventeen healthy male volunteers (28.9 ± 11.0 years, Table 1) participated in the experiments. No participant had a history of neurological or musculoskeletal injuries or diseases. All procedures were approved in advance by the local ethics committee of the Tokyo Metropolitan Institute of Medical Science (approval number 17-2) and conducted in accordance with the Declaration of Helsinki. Written informed consent was obtained from all participants prior to testing.

**TABLE 1:** Participants’ age, stimulus site and intensity, and participated experiments.

Participants	Age (years)	Site of coil upper edge	Stimulus intensity (% MSO)	Participated experiments		
1	48	L1–L2	10–60	Exp 1		
2	22	L1–L2	10–60	Exp 1		
3	22	L1–L2	10–70	Exp 1		
4	21	L1–L2	10–70	Exp 1		
5	49	L1–L2	10–70	Exp 1		
6	29	L1–L2	10–70	Exp 1	Exp 2	Exp 3
7	42	L2–L3	10–60	Exp 1	Exp 2	Exp 3
8	33	L1–L2	10–70	Exp 1	Exp 2	Exp 3
9	23	L2–L3	10–60	Exp 1	Exp 2	Exp 3
10	41	L1–L2	10–70	Exp 1	Exp 2	Exp 3
11	41	L1–L2	10–70	Exp 1	Exp 2	Exp 3
12	22	L1–L2	25, 55			Exp 3
13	19	–	–		Exp 2	
14	20	–	–		Exp 2	
15	20	–	–		Exp 2	
16	20	–	–		Exp 2	
17	19	–	–		Exp 2	

Exp 1, Experiment 1; Exp 2, Experiment 2; Exp 3, Experiment 3; MSO, maximum stimulator output; L1-L2, between 1st and 2nd lumbar vertebrae; L2-L3, between 2nd and 3rd lumbar vertebrae.

### Experimental setup

**FIGURE 1:**
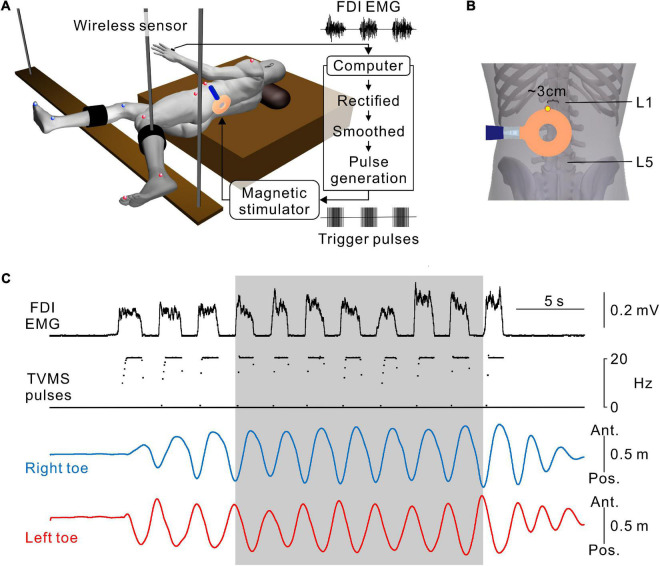
Hand muscle-controlled transvertebral magnetic stimulation (TVMS) to induce leg movements. **(A)** Schematic illustration of the experimental setup and apparatus. The participant was in a semi-prone position on a bed with the left side up. The right and left legs were secured on leg braces that were suspended by wires from the ceiling. Reflective markers for a three-dimensional motion capture system were placed on the shoulder, 6th rib under the arm, iliac crest, major trochanter, knee, malleolus, and toe on the left (red spheres), and major trochanter, knee, malleolus, and toe on the right (blue spheres). Electromyography (EMG) recorded from the first dorsal interosseous muscle (FDI) was transformed to TVMS pulses via a closed-loop algorithm. **(B)** An example of stimulus coil configuration. The upper edge of the round coil was set at an intervertebral space between L1 and L2 (yellow dot). **(C)** An example of walking-like movements evoked by TVMS at an intensity of 70% maximum stimulator output (MSO). According to the amplitude of the FDI EMG (1st row), the temporal profile of TVMS (2nd row) was controlled by the participant. Right and left toe movements (3rd and 4th rows) are presented on the antero-posterior axis. Seven continuous cycles of movements, in which the amplitude and cycle of leg movements became stable at least after the 3rd cycle from initiation, were used for further analysis (gray area).

The experimental apparatus has been described elsewhere (Selionov et al., 2009; Gerasimenko et al., 2010; Sasada et al., 2014). During the experiments, the participants were in a semi-prone position on a bed with the left side up (Figure 1A). Their legs were suspended by wires to keep the participants relaxed. This apparatus supported low-friction movements of the legs, so that the participants were able to readily perform leg movements in the horizontal plane. The participants were asked to keep their legs relaxed throughout the experiments and not to resist the movements induced by TVMS.

### Transvertebral magnetic stimulation

A full description of the method for TVMS has been published previously (Sasada et al., 2014). Briefly, stimulation over the lumbar vertebrae was applied using a magnetic stimulator with a circular coil with a diameter of 90 mm (Magstim Company Ltd., Whitland, UK). The upper edge of the circular coil was placed at the intervertebral region (Figure 1B). The tip of the circular coil was positioned such that the eddy current induced by magnetic stimulation entered the intervertebral space and flowed into the lower back of the participant in a counterclockwise direction from the examiner’s point of view.

### Closed-loop transvertebral magnetic stimulation protocol

A full description of the protocol for closed-loop TVMS has been published previously (Sasada et al., 2014; Kato et al., 2016, 2019). To induce rhythmic leg movements, a computer interface was used to control the temporal profiles of the TVMS triggers and to activate the spinal locomotor circuitry around the lumbar cord. Muscle activity was recorded from the first dorsal interosseous muscle during hand grip and converted to trigger pulses controlling the magnetic stimuli delivered over the lumbar vertebrae (Figures 1A,B). The computer interface was designed to encode the outline of full-wave rectified and moving averaged (250-ms window) surface electromyographic (EMG) activity from a muscle, and to convert the encoded EMG activity (X [a.u.]) into electrical rectangular pulses. The frequency of these pulses was determined by the level of EMG activity from the input muscle (Figure 1C). Using the output channels, the participants were able to alter the frequency of magnetic stimulation voluntarily through the interface. If input muscle activity (X [a.u.]) was above the stimulus threshold (X_th_ [Hz]), the frequency (f [Hz]) was modulated by the following equation:

$$f=f_{0}+\frac{f_{\text{g}}}{X_{t\,h}}\cdot X,\left(f\leq f_{M\,a\,x}\right)$$

where *f*_0_ = stimulation frequency at *X*_*th*_ [Hz], *f*_0_ = 2, *f*_*g*_ = stimulation frequency gain, *f*_*Max*_ = maximum stimulation frequency [Hz], *f*_*Max*_ = 20.

Before each session, we measured the background noise level and the amplitude of the input EMG activity; then, *X*_*th*_ and *f*_*g*_ were set arbitrarily by the experimenter. *X*_*th*_ was set as a value at which muscle activity could be detected without contamination with background signal noise and stimulus artifacts. Stimulus frequency gain (*f*_*g*_) was also set as a value at which *f*_*Max*_ was obtained at the peak amplitude of input EMG activity when the participants performed hand gripping with their comfortable strength.

### Experimental protocols

Three experiments were conducted in subgroups of the participants: Experiment 1 for evoked movements (*n* = 11), Experiment 2 for voluntary movements (*n* = 11), and Experiment 3 for evoked movements with the restriction of lumbar spine movement (*n* = 6). Six out of 17 participants were tested in all experiments; five participants were tested only in Experiment 1; another five participants were tested only in Experiment 2; and one participant was examined only in the Experiment 3 (see Table 1).

#### Experiment 1

Prior to obtaining data, we determined the optimal site for evoking walking-like movements in each participant (Figure 1B). We identified the intervertebral spaces by palpation. Stimulus intensity was kept constant in each participant, and its range was set at 40–60% of the maximum stimulator output (MSO) of the magnetic stimulator. Stimulation was applied at one intervertebral space (L1–L2 or L2–L3). The lateral position was shifted approximately 3 cm to the left from the midline if needed. After that, while the participants relaxed their legs, we applied TVMS at seven intensities (10%, 20%, 30%, 40%, 50%, 60%, and 70% MSO) in a randomized order across participants. The participants performed rhythmic hand gripping with obtaining visual feedback of their leg movement to deliver the stimuli for 1–2 min, allowing us to collect ∼20 continuous cycles at each intensity. Four participants felt pain when stimulation was applied at 70% MSO, and thus 70% MSO was not assessed further in these participants (Table 1).

#### Experiment 2

Under the same experimental setting, the participants were asked to perform voluntary hopping and walking leg movements. For hopping movements, the participants were asked to swing both legs back and forth in-phase. For the walking movements, the participants were asked to perform alternating bilateral leg swings in anti-phase. The participants performed both movements for ∼20 cycles each with verbal feedback from the experimenters to maintain the same movement rhythms as in Experiment 1. On the basis of the results of Experiment 2 (see section “Results” and Figure 3), the evoked movements were categorized as hopping, walking, or uncategorized.

**FIGURE 2:**
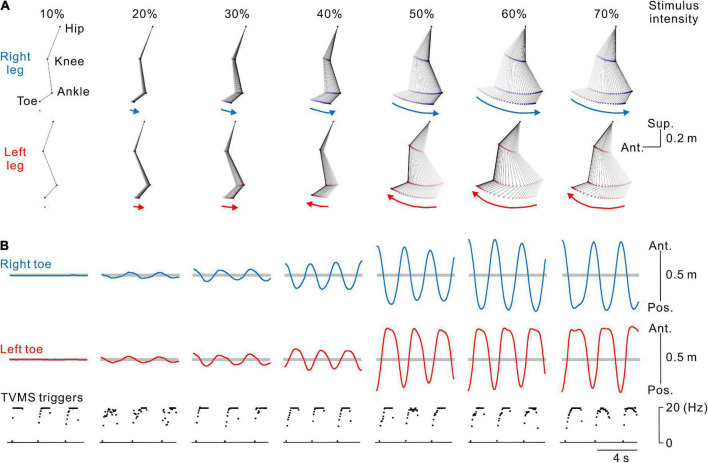
Kinematics of stimulus-evoked leg movements. **(A)** Stick pictures showing examples of right (blue) and left (red) leg movements evoked by transvertebral magnetic stimulation (TVMS) at different stimulus intensities in a single participant. From left to right, stimulus intensity of 10% to 70% maximum stimulator output (MSO). Arrows below the stick pictures indicate the direction of the induced leg movement at the timing of the stimulus trains. **(B)** Traces showing the movement trajectories of the right (blue lines in the top row) and left (red lines in the middle row) toes in the antero-posterior axis and spinal stimulation (bottom row). Horizontal gray areas behind the toe movement traces represent the ranges between ± 5 standard deviations of toe movements calculated during the baseline period of 2 s before the beginning of TVMS.

**FIGURE 3:**
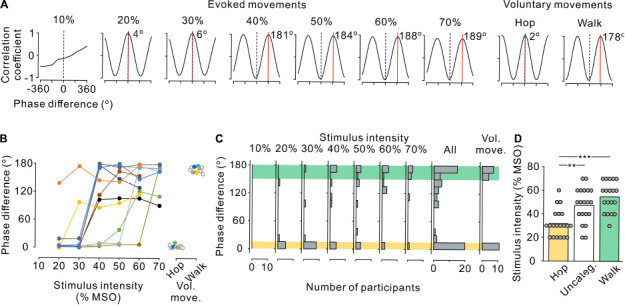
Phase difference between left and right leg movements. **(A)** Cross-correlograms of left and right leg movements at the anterior-posterior axis in a single participant during stimulus-evoked movements (left seven panels) and voluntary movements (right two panels). Phase difference between the left and right step cycles was estimated by the time shift of the peak coefficient of correlation multiplied by the fundamental angular frequency. Red line and inset number in each correlogram indicate the phase shift on the positive peak. Note that a positive (right) shift indicates the leading of left movements with reference to right movements. **(B)** Phase difference between left and right leg movements during stimulus-evoked movements at different stimulus intensities (left) and voluntary movements (right) in all participants. Colored symbols and lines represent data for each individual participant. Open circles represent the participants in whom only voluntary movements were measured (right panel). Note that the absolute values of phase differences were transformed to a range of 0–180°. **(C)** Histograms of the number of participants with leg movements at different left-right phases. From left to right, the stimulus-evoked leg movements at 10–70% maximum stimulator output (MSO) and all intensities combined and voluntary leg movements. Yellow and green hatched areas indicate the range of phase difference obtained during the voluntary hopping (0–15°) and walking (150–180°) movements, respectively. **(D)** Stimulus intensity during the three patterns of stimulus-evoked leg movements defined based on the range of voluntary hopping and walking movements: hopping-like (hop, 0–15°, yellow), uncategorized (Uncateg., 15–150°, white), and walking-like (walk, 150–180°, green). Gray circles represent individual participants at all stimulus conditions. Significant differences were revealed by *post hoc* multiple comparisons following analysis of variance (**p < 0.01 and ***p < 0.001).

#### Experiment 3

**FIGURE 4:**
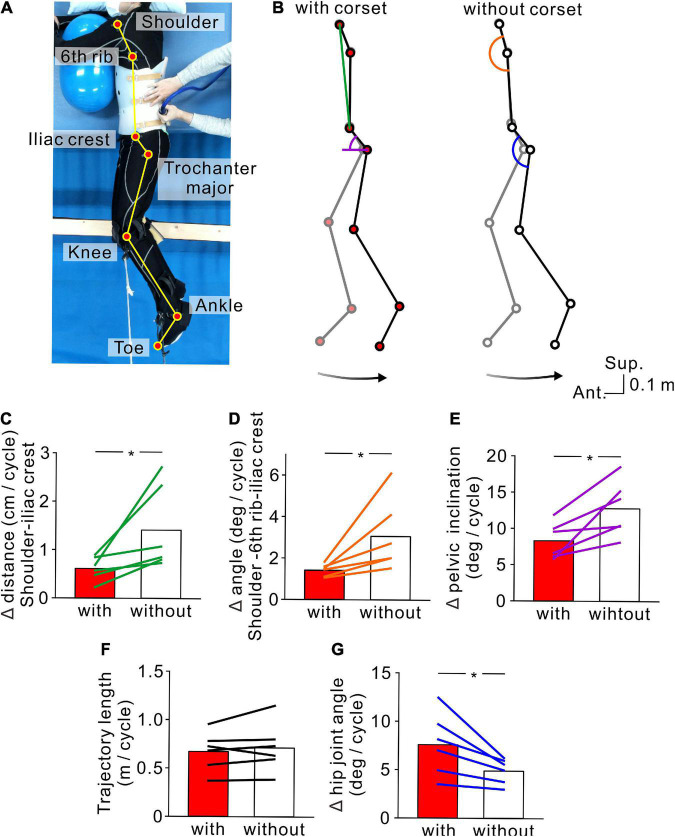
Kinematics of stimulus-evoked hopping-like leg movements. **(A)** Participant posture and reflective markers for three-dimensional motion capture. **(B)** Stick pictures showing examples of left leg movements evoked by transvertebral magnetic stimulation in the conditions with (left) and without (right) the lumbar corset. Note that the stick pictures represent the foremost (gray line) and rearmost (black line) positions of the left toe in a cycle. Colored line and semicircles indicate the measures for lumbar spine movements (green line = distance between the left shoulder and iliac crest, orange semicircle = angle formed by the left shoulder and 6th rib under the arm and iliac crest, purple semicircle = pelvic inclination angle against the antero-posterior axis, and blue semicircle = hip angle). Bottom arrows show the toe trajectory of the above stick pictures. Population data (*n* = 6) of the distance between the left shoulder and iliac crest **(C)**, angle formed by the left shoulder and 6th rib under the arm and iliac crest **(D)**, pelvic inclination against the antero-posterior axis **(E)**, toe trajectory length **(F)**, and hip joint angle **(G)**. Red and white boxes represent the values in the conditions with and without the corset, respectively. Lines over the boxes represent individual participants. Significant differences were revealed by paired *t*-tests (*p < 0.05).

In order to clarify that TVMS was capable of evoking hopping-like leg movements without the flexion-extension movements of the lumbar spine, as a control experiment, we tested six participants while they wore a lumbar plastic corset to restrict flexion-extension movements of the lumbar spine (Figure 4A). The transparent material of the corset allowed us to place the magnetic coil over the vertebrae, similar to the condition without the corset. TVMS was delivered with same protocol in Experiment 1. To validate coil position, we confirmed that high-intensity TVMS evoked walking-like movements before testing. Then, stimulus intensity was decreased until they transformed into hopping-like movements (47.5 ± 10.4% MSO). To evaluate the restriction of lumbar spine movements, we also delivered TVMS over the same vertebral level after the participant took off the corset. Then, the kinematics of pelvic movements were compared across conditions with and without the corset. As it was impossible to keep the distance between the stimulus coil and the surface of the back skin identical across conditions, we adjusted stimulus intensity in the condition without the corset (27.8 ± 8.7% MSO) to match the amplitude of evoked leg movements with that in the condition with the corset.

### Data recording

EMG of the first dorsal interosseous muscle used for controlling TVMS was recorded with wireless EMG sensors (Trigno™; Delsys Co., Ltd., Natick, MA, USA). In addition, EMG signals were recorded from the iliopsoas, gluteus maximus, rectus femoris, vastus lateralis, biceps femoris, tibialis anterior, and soleus muscles on both sides. EMG signals were amplified and bandpass filtered at 20–450 Hz. EMG signals and transistor-transistor logic pulses triggering the stimulator were converted to digital data *via* an A/D converter system at a sampling rate of 5 kHz for later off-line analysis (CED 1401 interface with Spike2 software; Cambridge Electronic Design Ltd., Cambridge, UK).

Leg movements were measured using eight infrared cameras in a three-dimensional motion capture system (Flex3; OptiTrack, Inc., Corvallis, OR, USA). Positional changes of the legs were detected by reflective markers on the major trochanters (Hip), points between the femoral and tibial condyles (Knee), malleolus (Ankle), and toes (Toe) of both legs (Figures 1A, 2A). For only Experiment 3, we additionally recorded the positions of the shoulder, 6th rib under the arm, and iliac crest on the left side (Figures 4A,B). Recorded data were stored on a computer at a recording frequency of 100 Hz and were used for offline analysis.

### Analysis of leg movements and muscle activity

#### Leg kinematics

The participants were asked to received voluntarily controlled stimulation (Experiments 1, 3) or perform volitionary leg movements (Experiment 2) for ∼20 cycles. Seven continuous cycles of movements, in which the amplitude and cycle of leg movements became stable at least after the 3rd cycle from initiation, were used for analysis (Figure 1C). First, we calculated the average and standard deviation (SD) of bilateral toe positions in the antero-posterior axis for 2 s prior to stimulation in each session. Then, any movements that occurred within the threshold range (±5 SD from the mean, Figure 2B) were defined as a state in which no movement was induced by stimulation (e.g., Figures 2A,B, 10% MSO). Stimulus-evoked movements were defined when bilateral toe movements exceeded the threshold range (e.g., Figures 2A,B, ≥20% MSO). Then, only data for the detected evoked movements were used for further analysis. To characterize the evoked movements, we employed cross-correlation analysis to estimate the phase difference between the left and right toe movement cycles (Figure 3A). For the sake of categorization of leg movement type, the absolute values of phase difference (θ_*abs*_ [°]) below 0° and above 180° were transformed to a range of 0°−180° (θ_*trans*_ [°]) by the following equation:

$$\theta_{t\,r\,a\,n\,s}=|\theta_{a\,b\,s}|,\left(\theta_{a\,b\,s}<0\right)$$

$$\theta_{t\,r\,a\,n\,s}=360-\theta_{a\,b\,s},\left(\theta_{a\,b\,s}>180\right)$$

**FIGURE 5:**
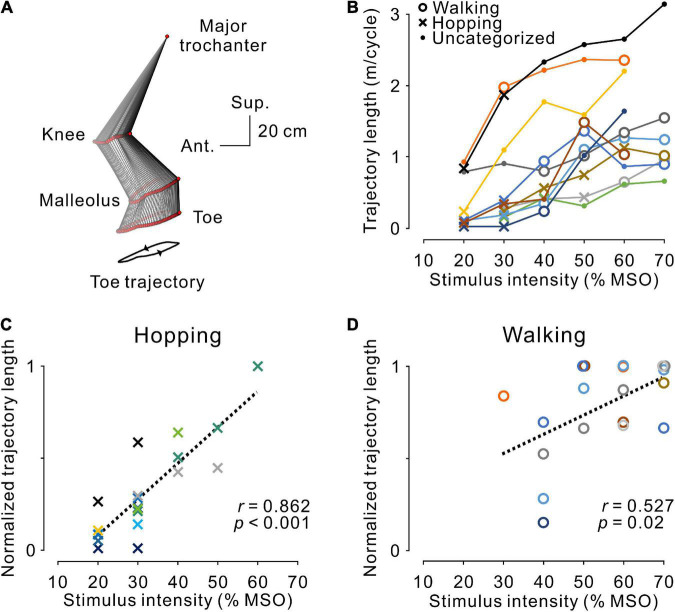
Magnitude of stimulus-evoked leg movements. **(A)** An example of leg movements on the left side. The line with two arrowheads under the stick picture illustrates the toe trajectory. **(B)** The magnitude of stimulus-evoked leg movements at different stimulus intensities. Plotted data were obtained from the averaged toe trajectories in seven sequential steps. Open circles, dots, and crosses indicate hopping-like, uncategorized, and walking-like movements, respectively, as defined in Figure 3D. The relationship between stimulus intensity and the magnitude of the evoked hopping-like **(C)** and walking-like **(D)** movements. Toe trajectory length was normalized by the maximum value of each participant. Note that a linear relationship was observed during both movements (hopping-like, *r* = 0.923, *p* < 0.001; walking-like, *r* = 0.527, *p* = 0.020; Pearson’s correlation analysis). The color code of each participant is the same as in Figure 3.

To quantify the magnitude of the stimulus-evoked movements, the length of two-dimensional (antero-posterior and superior-inferior) toe displacement trajectory was analyzed for seven sequential cycles (Figure 5). For Experiment 3, lumbar spine movements were evaluated with the following measures obtained in seven stable continuous cycles: distance between the left shoulder and iliac crest, angle formed by the shoulder, 6th rib under the arm, and iliac crest, and the pelvic inclination angle against the antero-posterior axis (Figure 4B). Leg movements were measured by hip angle and toe trajectory length (Figure 4B).

#### Muscle activity

**FIGURE 6:**
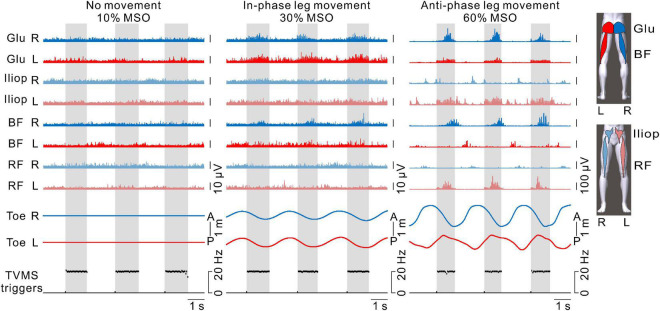
The activation patterns of hip flexor and extensor muscles in both legs. From left to right, examples with transvertebral magnetic stimulation (TVMS) at 10%, 30%, and 60% maximum stimulator output (MSO). The blue and red traces represent electromyography (EMG) responses in the right and left leg muscles, respectively. The traces with light and dark colors represent EMG responses in the anterior (iliopsoas [Iliop], rectus femoris [RF]) and posterior (gluteus maximus [Glu], biceps femoris [BF]) muscles, respectively. Note that stimulus artifacts were removed from the EMG traces by flattening for 5 ms from the stimulus triggers. Right and left toe movements and the TVMS triggers are shown as in Figure 2B. Gray hatched areas indicate the periods of TVMS trains.

To investigate the pattern of muscle activity, bilateral leg EMGs were rectified throughout the experiments (Figure 6). To remove stimulus artifacts, the EMG traces were flattened for 5 ms after the stimulus trigger.

**FIGURE 7:**
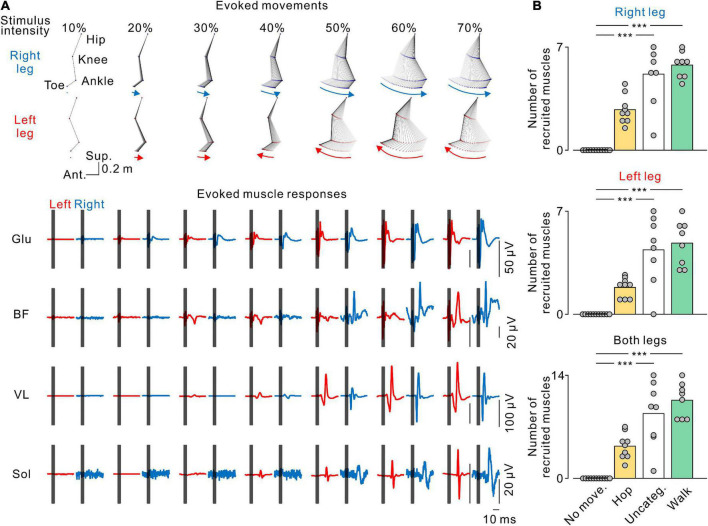
Stimulus-evoked electromyography (EMG) responses in leg muscles. **(A)** The stick pictures show the evoked leg movements and are the same as in Figure 2A. The traces show the stimulus-evoked EMG responses in the gluteus maximus (Glu), biceps femoris (BF), vastus lateralis (VL), and soleus (Sol) muscles in the left (red) and right (blue) legs at each stimulus intensity. Dark gray areas in the EMG traces hide stimulus artifacts. **(B)** Number of muscles recruited by transvertebral magnetic stimulation. From top to bottom, the number of recruited muscles in the right, left, and both legs at each pattern of leg movement. No movement (No move., black), hopping-like movements (Hop, yellow), uncategorized movements (Uncateg., white), and walking-like movements (Walk, green) were defined by the same criteria as in Figures 1, 3. The values of the bars were averaged in each participant. Circles indicate the results of each participant. Significant differences were revealed by Dunn’s tests following Kruskal−Wallis one-way analysis of variance (***p < 0.001).

To identify whether the recorded muscles were activated by TVMS, we averaged each raw EMG trace with respect to the TVMS trigger for seven continuous movement cycles. Then, the mean and SD of background EMG were measured in a 20-ms window prior to the stimulus trigger. Muscle recruitment by TVMS was determined when the averaged EMG traces exceeded the threshold level (mean + 2 SD) for at least 5 ms within 5–50 ms after the TVMS trigger. After that, we counted the number of recruited muscles in each leg and stimulus intensity (Figure 7).

### Statistical analysis

Data normality was tested by the Shapiro−Wilk test. When normality was not assumed, a non-parametric test was conducted on rank-ordered data. To clarify the uniformity of TVMS triggers across stimulus intensities, we conducted either parametric one-way analysis of variance (ANOVA) or Kruskal−Wallis one-way ANOVA on the temporal profiles of TVMS (train duration, number of pulses per train, mean stimulus frequency, and inter-train interval). One-way ANOVA followed by Bonferroni-corrected multiple *t*-tests was conducted to determine the significant differences in stimulus intensity across the evoked movements (hopping-like, uncategorized, and walking-like movements). We also conducted the Mann−Whitney *U*-test to compare the magnitude of the evoked hopping- and walking-like movements. Pearson correlation coefficients were computed between stimulus intensity and normalized trajectory length in the evoked hopping- and walking-like movements, respectively. Kruskal−Wallis one-way ANOVA followed by multiple comparisons with Dunn’s test was conducted to compare the number of recruited leg muscles between no movement and the evoked rhythmic leg movements (hopping-like, uncategorized, walking-like movements). For data in Experiment 3, we conducted paired *t*-tests to compare across conditions (with corset, without corset). Statistical significance was set at alpha <0.05. Population data are presented as the mean ± SD in the text.

## Results

We investigated the relationship between the intensity of magnetic stimulation applied to lumbar vertebrae and stimulus-evoked leg movements in 11 healthy participants. The optimal site for evoking walking-like movements was at the intervertebral space of L1–L2 or L2–L3 (Figure 1B and Table 1, Stimulus site). Stimulus intensity from 10 to 70% MSO was investigated in seven participants, while stimulus intensity from 10 to 60% MSO was investigated in the remaining four because of their discomfort with high stimulus intensity (Table 1). No participant showed visible rhythmic leg movements with TVMS at 10% MSO. At 20% MSO, bilateral rhythmic leg movements were induced in 8 of 11 participants. At an intensity not less than 30% MSO, all 11 participants exhibited clearly identifiable bilateral rhythmic leg movements. The temporal profiles of TVMS triggers were demonstrated to be similar across stimulus intensities (Table 2; Train duration, *X*^2^ = 4.822, *p* = 0.567; Number of pulses per train, *X*^2^ = 4.381, *p* = 0.625; Mean stimulus frequency, *X*^2^ = 0.963, *p* = 0.987, Kruskal−Wallis one-way ANOVA; Inter-train interval, *F_6_,_66_* = 0.158, *p* = 0.987, one-way ANOVA).

**TABLE 2:** Temporal profiles of stimulus triggers in Experiment 1.

Stimulus intensity (% MSO)	Train duration (s)	Number of pulses/train	Mean stimulus frequency (Hz/train)	Inter-train interval (s)
10	1.21 ± 0.22	23.3 ± 4.6	18.4 ± 0.7	1.29 ± 0.24
20	1.23 ± 0.26	23.5 ± 5.0	18.4 ± 1.0	1.32 ± 0.26
30	1.23 ± 0.23	23.8 ± 4.8	18.5 ± 0.7	1.36 ± 0.28
40	1.19 ± 0.24	22.9 ± 5.2	18.4 ± 1.0	1.34 ± 0.17
50	1.14 ± 0.25	21.9 ± 5.1	18.2 ± 0.8	1.30 ± 0.19
60	1.06 ± 0.23	20.4 ± 4.7	18.3 ± 0.8	1.33 ± 0.18
70	1.24 ± 0.19	23.9 ± 3.2	18.5 ± 0.7	1.36 ± 0.20
	*p* = 0.567	*p* = 0.625	*p* = 0.987	*p* = 0.987

MSO, maximum stimulator output.

### Effect of stimulus intensity on the pattern of stimulus-evoked leg movements

Figure 2 show typical examples of the bilateral leg movements evoked by TVMS applied to the lumbar vertebrae at seven stimulus intensities. The amplitude of the evoked leg movements increased in a stimulus intensity-dependent manner. In these examples, no obvious leg movements were observed at 10% MSO (see also Supplementary Video 1). At 20% and 30% MSO (see also Supplementary Video 2), bilateral in-phase leg movements were induced. The backward movement was in the rearmost position when stimulation frequency was approximately at its peak, while the forward movement was in the foremost position when stimulation reached its nadir. However, at stimulus intensities greater than 40% MSO, stimulation evoked alternative left-right leg movements, as in walking (see also Supplementary Video 3).

To characterize the stimulus-evoked leg movements, the phase difference between left and right movements was estimated using cross-correlation analysis. Figure 3A shows representative cross-correlograms of toe movement patterns in a single participant during evoked and voluntary movements. The red line and inset number in each correlogram indicate the phase difference of the right (reference) and left (analysis) toes. At 20% and 30% MSO, the phase differences were close to 0°, indicating that both toes moved in the same direction in phase, as in hopping. Conversely, at ≥40% MSO, the phase differences were close to 180°, indicating that the toes moved alternatively in anti-phase, as in walking. To compare the phase difference between the evoked and voluntary leg movements, the participants were asked to perform hopping and walking movements voluntarily in the Experiment 2. The phase difference in voluntary hopping was close to 0°, which was identical to stimulus-evoked movements at 20% and 30% MSO. Similarly, the phase difference in voluntary walking was close to 180°, which was identical with stimulus-evoked movements at ≥40% MSO.

We investigated the effect of stimulus intensity on the phase difference between left and right toe movements in all 11 participants (Figure 3B). The phase difference showed two major distributions, one was at approximately 180° and the other was close to 0°. On the basis of the results of Experiment 2 for voluntary movements, the left-right phase difference in voluntary hopping and walking was distributed at <15° and >150°, respectively (Figures 3B,C). Thus, the evoked movements were defined as hopping-like movements when the phase difference of the stimulus-evoked leg movements was within the range of voluntary hopping (≤15°) and as walking-like movements when the phase difference was within the range of voluntary walking (≥150°) (Figure 3C). Stimulus-evoked movements that fulfilled neither of these conditions were defined as uncategorized movements (>15°–<150°, Figure 3C). According to this definition, hopping-like movements were induced in 9 of 11 participants, and walking-like movements were induced in 8 of 11 participants (Figure 3B). From the view point of stimulus intensity, hopping-like movements were evoked in 6 of 8 participants (75.0%) at 20% MSO, 8 of 11 participants (72.7%) at 30% MSO, 3 of 11 participants (27.3%) at 40% MSO, 2 of 11 participants (18.2%) at 50% MSO, 1 of 11 participants (9.1%) at 60% MSO, and 0 of 7 participants (0%) at 70% MSO. In contrast, walking-like movements were evoked in 0 of 7 participants (0%) at 20% MSO, 1 of 11 participants (9.1%) at 30% MSO, 4 of 11 participants (36.4%) at 40% MSO, 4 of 11 participants (36.4%) at 50% MSO, 5 of 11 participants (45.5%) at 60% MSO, and 5 of 7 participants (71.4%) at 70% MSO (Figure 3C).

The stimulus-evoked leg movements in 5 of 11 participants changed from hopping-like movements into walking-like movements directly as stimulus intensity increased (Figure 3B). Hopping-like movements were not observed in two participants; however, the evoked movements changed from uncategorized movements to walking-like movements in these individuals (Figure 3B). Walking-like movements were not observed in the other three participants; however, the hopping-like movements transformed into uncategorized movements in these individuals as stimulus intensity increased (Figure 3B). In only one participant, the evoked leg movements changed sequentially from hopping-like movements to uncategorized movements and then to walking-like movements as stimulus intensity increased (Figure 3B). The stimulus intensity required to evoke walking-like and uncategorized leg movements was higher than that for evoking hopping-like movements (Figure 3D, *F_2_,_56_* = 15.496, *p* < 0.001, one-way ANOVA; hopping-like vs. uncategorized, *t* = 3.750, *p* = 0.001; Hop vs. Walk, *t* = 5.429, *p* < 0.001; Bonferroni corrected multiple *t*-tests). Thus, low stimulus intensity was more likely to induce hopping-like movements, and high stimulus intensity was more likely to induce walking-like movements.

In order to confirm that the hopping-like movements certainly engaged the leg movements, we tested six participants in which flexion-extension movements of the lumbar spine were restricted by a lumbar corset (Experiment 3, see section “Materials and methods”). We found that the TVMS-evoked hopping-like movements were preserved even when lumbar spine movements and pelvic tilt were restricted. Regardless of conditions with or without the lumbar corset, TVMS evoked hopping-like leg movements in which the phase difference between the left and right leg movement cycles was less than 15° in all participants (with corset, 2.64 ± 4.25°; without corset, 2.50 ± 3.34°; *t* = 0.0716, *p* = 0.946, paired *t*-test). In spite of the similar magnitude of in-phase hopping-like leg movements, the difference in pelvic motion was noticeable across conditions (Figure 4B). In the conditions with the corset, the changes in the distance between the left shoulder to iliac crest (Figure 4C, *t* = −2.581, *p* = 0.0493, paired *t*-test) and the angle formed by the positions of the left shoulder and 6th rib under the arm and iliac crest (Figure 4D, *t* = −2.706, *p* = 0.042, paired *t*-test) were significantly smaller compared with the condition with the corset. More directly, we also observed that the pelvic inclination angle against the antero-posterior axis changed less in the presence of the corset than without corset (Figure 4E, *t* = −3.460, *p* = 0.018, paired *t*-test). In the condition with the corset, the same magnitude of hopping-like leg movements (Figure 4F, *t* = −1.100, *p* = 0.321, paired *t*-test) was compensated for by larger hip joint movements (Figure 4G, *t* = 3.145, *p* = 0.026, paired *t*-test). We also confirmed that the temporal profiles of TVMS were similar across conditions (Table 3; Train duration, *t* = 0.363, *p* = 0.732; Number of pulses per train, *t* = 0.327, *p* = 0.757; Mean stimulus frequency, *t* = −0.740, *p* = 0.492; Inter-train interval, *t* = −2.174, *p* = 0.082; all by paired *t*-test).

**TABLE 3:** Temporal profile of stimulus triggers in Experiment 3.

Experimental condition	Train duration (s)	Number of pulses/train	Mean stimulus frequency (Hz/train)	Inter-train interval (s)
With corset	0.95 ± 0.11	19.1 ± 2.1	18.9 ± 0.2	1.43 ± 0.18
Without corset	0.93 ± 0.16	18.7 ± 3.2	18.9 ± 0.2	1.57 ± 0.16
	*p* = 0.732	*p* = 0.757	*p* = 0.492	*p* = 0.082

### Effect of stimulus intensity on the amplitude of evoked leg movements

As observed in the representative example shown in Figure 2, regardless of the pattern of stimulus-evoked leg movements, their amplitude seemed to depend on stimulus intensity and became larger as intensity increased. This tendency corresponded with the population data shown in Figure 4B. Accordingly, in general, the magnitude of leg movements, measured by toe trajectory length, was larger in the walking-like movements (*n* = 8, 1.10 ± 0.53 m/cycle) than in the hopping-like movements (*n* = 9, 0.43 ± 0.44 m/cycle, *U* = 11.000, *p* = 0.016, Mann−Whitney *U*-test). We investigated the effect of stimulus intensity on the trajectory length of toe movements in hopping-like (Figure 5C) and walking-like (Figure 5D) movements. For linear regression analysis on all participants’ data, toe trajectory length was normalized by the maximum value in each participant. We found a positive correlation between stimulus intensity and normalized toe trajectory length in the hopping-like (*n* = 20, *r* = 0.862, *p* < 0.001, Pearson correlation analysis, Figure 5C) and walking-like (*n* = 19, *r* = 0.527, *p* = 0.0204, Pearson correlation analysis, Figure 5D) movements.

### Effect of stimulus intensity on muscle recruitment during stimulus-induced leg movements

Figure 6 shows the activation patterns in the bilateral hip flexors and extensors in a representative participant. No obvious EMG activity was observed when TVMS applied at 10% MSO did not evoke rhythmic leg movements. When in-phase leg movements were evoked at 30% MSO, bilateral activation appeared in the hip extensor muscles (gluteus maximus and biceps femoris) during the TVMS trains. In contrast, when the evoked leg movements were anti-phase at 60% MSO, an alternative activation pattern was observed between the interlimb leg muscles as well as between intralimb flexor and extensor muscles. During the TVMS trains, the hip flexors (gluteus maximus and biceps femoris muscles) exhibited dominant activation in the left leg, whereas the hip extensors (iliopsoas and rectus femoris muscles) exhibited dominant activation in the right leg. In addition, those muscle activation patterns were switched to antagonistic muscle activity (iliopsoas and rectus femoris muscles in the right leg, biceps femoris muscle in the left leg) between the TVMS trains. We also observed co-activation in the left gluteus maximus and iliopsoas muscles during the TVMS trains. In all participants, the higher the stimulus intensity was, the more often the co-activation pattern was evoked in the intralimb flexor and extensor muscles, even if the evoked leg movements were anti-phase (data not shown).

We found that increasing stimulus intensity changed the pattern (Figure 4) and amplitude (Figure 5) of the evoked leg movements. These changes may reflect muscle recruitment. To elucidate the relationship between stimulus-evoked leg movements and the pattern of muscle recruitment, we investigated the recruitment of leg muscles during each evoked movement pattern. Figure 6A shows a representative example of the effect of stimulus intensity on the evoked movement pattern (same as in Figure 2A) and the evoked responses in the bilateral leg muscles. At the stimulus intensity which did not evoke any rhythmic leg movements, no evoked responses were observed in any leg muscle (e.g., Figure 7A, 10% MSO). However, once either pattern of rhythmic movement was evoked, all participants exhibited short latency-evoked responses in some leg muscles (e.g., Figure 7A, ≥20% MSO). Comparing across the evoked movement patterns, we observed a trend that the number of recruited muscles gradually increased from the absence of movement to the hopping-like movements, uncategorized movements, and walking-like movements (right leg, *H* = 28.988, *p* < 0.001; left leg, *H* = 27.264, *p* < 0.001; both legs, *H* = 29.003, *p* < 0.001; all by Kruskal−Wallis one-way ANOVA; Figure 6B). More muscles were recruited during the uncategorized and walking-like movements than during no movement (Uncateg. vs. No move., right leg, *p* < 0.001, left leg, *p* < 0.001, both legs, *p* < 0.001; Walk vs. No move., right leg, *p* < 0.001, left leg, *p* < 0.001, both legs, *p* < 0.001; all by Dunn’s test). However, there was no difference in the number of recruited muscles between the hopping-like movements and the other movement types (Hop vs. No move., right leg, *p* = 0.103, left leg, *p* = 0.141, both legs, *p* = 0.094; Hop vs. Uncateg., right leg, *p* = 0.336, left leg, *p* = 0.467, both legs, *p* = 0.568; Hop vs. Walk, right leg, *p* = 0.170, left leg, *p* = 0.151, both legs, *p* = 0.119; all by Dunn’s test).

## Discussion

We demonstrated that TVMS delivered to the human lumbar spinal cord in a closed-loop manner evoked multi-patterned locomotion-like rhythmic leg movements, and that the movement pattern transformed depending on the intensity of TVMS. At a low stimulus intensity slightly above the motor threshold of the leg muscles (20–30% MSO), hopping-like movements were induced in which the left and right legs moved in the same direction in phase. When stimulus intensity was increased, the phase between the left and right movements changed to out-of-phase; in most cases, the left and right legs moved in opposite directions as walking-like movements. These findings indicate that TVMS may activate the distinct spinal neural modules responsible for generating hopping- and walking-like leg movements in humans.

### Transvertebral magnetic stimulation activates spinal locomotor circuitry

The current study confirmed previous findings that percutaneously delivered repetitive TVMS targeting the lumbar spinal cord in humans induced alternating bilateral leg movements, similar to walking (Gerasimenko et al., 2010; Sasada et al., 2014). It is still unclear which neuronal elements are driven by TVMS. TVMS most likely activates nerve fibers at the roots non-selectively through induced eddy currents (Ugawa et al., 1989; Fujishiro et al., 2000; Matsumoto et al., 2013) and indirectly activates spinal circuits. We assume that repetitive TVMS drives such an indirect spinal neural network which is capable of producing alternating activation of the left-right homologous muscles and intralimb flexor-extensor muscles. A locomotor central pattern generator has been identified in the mammalian spinal cord (for reviews see, Grillner, 1981; Kiehn, 2006) and can be activated by electrical stimulation of dorsal roots to generate walking movements (Barthélemy et al., 2006, 2007). These findings have also been confirmed in humans. Experiments in paralyzed individuals with SCI demonstrated that tonic epidural electrical stimulation of the dorsal aspect of the lumbosacral spinal cord induces rhythmic bilateral leg movements accompanied with corresponding rhythmic leg muscle activity (Dimitrijevic et al., 1998; Minassian et al., 2004), indicating that the human lumbosacral spinal cord possesses central pattern generator-like locomotor circuitry which can be driven by external artificial stimulation. We confirmed that percutaneous magnetic stimulation of the lumbar cord also evoked rhythmic leg muscle activity which consequently produced walking-like bilateral leg movements.

The induction of walking-like movements by tonic TVMS was reported to be limited to a small population (∼10%) of intact human subjects (Gerasimenko et al., 2010). However, our previous and present studies have improved the efficacy to induce walking-like movements by using periodic trains of TVMS, the temporal profiles of which were controlled by a closed-loop algorithm (Sasada et al., 2014; Kato et al., 2016, 2019). Indeed, in the present study, our stimulus paradigm enabled the induction of rhythmic out-of-phase bilateral leg movements in all 11 participants (Figure 3). Consistent with our findings, experiments in spinalized cats demonstrated that periodic trains of spinal stimulation are more effective than tonic spinal stimulation for the induction of bilateral hindlimb locomotion (Barthélemy et al., 2007). Speculatively, periodical TVMS trains might be close to the optimal input for the spinal locomotor circuitry to generate walking-like bilateral leg movements.

### Distinct neural modules for walking-and hopping-like movements

The most intriguing finding of this study was that rhythmically controlled TVMS evoked bilateral in-phase leg movements as well as bilateral anti-phase leg movements, as hopping and walking, respectively, at different stimulus intensities. Threshold intensity to evoke each type of leg movement was variable across participants which was presumably due to the differences in physical size and body composition. However, it is consistent that low-intensity stimulation was more likely to evoke hopping-like movements, whereas high-intensity stimulation was more likely to evoke walking-like movements (Figures 2, 3, 5, 6). A reasonable explanation for this discrepancy is that low- and high-intensity TVMS activate distinct neuromuscular elements that have different activation thresholds, producing the different patterns of bilateral rhythmic leg movements. Theoretically, TVMS could activate not only the anterior and posterior nerve roots and intraspinal neurons but also the cutaneous receptors on the skin and muscles of the back under the stimulus coil (Fernandes et al., 2020). It is not convincing to state that the contraction of the back muscles generating the extension of the lumbar spine and the anterior tilt of the pelvis accounts for the anti-phase leg movements in the walking-like movements. Conversely, it is difficult to rule out the contribution of the contraction of the back muscles to the bilateral in-phase leg movements in the hopping-like movements. Nevertheless, the following two findings indicate that the rhythmic activation of leg muscles was certainly engaged in the production of the hopping-like movements. First, the rhythmic leg movements could not be detected without stimulus-evoked leg muscle responses (Figure 7). Second, the evoked hopping-like movements were preserved even when lumbar spine movement and anterior pelvic tilt were restricted by a lumbar corset (Figure 4). Given these facts, we favor the hypothesis that TVMS primarily activates neural elements around the lumbar spinal cord to evoke rhythmic leg movements, and that the activation of distinct neural modules is responsible for the generation of the hopping- and walking-like movements.

Low-intensity magnetic stimulation over vertebrae excites the low threshold large diameter afferents in the dorsal roots (Zhu et al., 1992). Therefore, which movement patterns are evoked might be determined by the activation threshold at the network level. Our findings suggest that human spinal neural circuits may require considerably more inputs to generate walking-like movements than the activation of spinal motoneuron pools for hopping-like movements, which is supported by an animal model showing that the intensity of spinal electrical stimulation required to induce bilateral hindlimb locomotion is higher than the motor threshold of muscle twitches (Barthélemy et al., 2007). Previous studies have demonstrated that the spinal locomotor neural circuitry hierarchically coordinates locomotor rhythm and left-right alternation (for reviews see, Kiehn, 2006, 2016), and that both types of coordination are controlled independently by spinal interneurons (Crone et al., 2008, 2009; Kiehn et al., 2008; Restrepo et al., 2011; Zhong et al., 2011; Talpalar et al., 2013; Pocratsky et al., 2017). Experiments using *in vivo* animal models have shown that inactivation of those interneurons transforms the hindlimb coordination pattern from walking to hopping (Talpalar et al., 2013; Pocratsky et al., 2017). Furthermore, the deprivation of left-right alternating limb coordination preserves intralimb coordination (Pocratsky et al., 2017), supporting the concept that the hopping and walking patterns of limb coordination are mediated by distinct neural modules.

The left-right limb coordination pattern in quadrupeds is known to transform sequentially from asymmetric to symmetric, such as walking to trotting followed by galloping and bounding, when locomotor speed increases (Forssberg et al., 1980; Hoyt and Taylor, 1981; Clarke and Still, 1999). Although the transformation of the limb coordination pattern can also be observed when changing from walking to running during human bipedal locomotion, both movements comprise alternating leg coordination patterns. Humans do not usually ambulate with bilateral hopping, which is presumably due to postural constraints on bipedal upright locomotion. Thus, it is not known if there is a neural circuitry in the human lumbar spinal cord specific for hopping. Besides, we need to acknowledge that the successive execution and cessation of the contraction of hip extensors by TVMS trains may underlie the hopping-like movements observed in this study. This is supported by the fact that the hopping-like movements were observed in the absence of substantial hip flexor activation (Figure 6, middle). Further investigations are required to determine the mechanisms of the spinal neural module responsible for the TVMS-evoked hopping-like movements.

### Methodological considerations

The current study tested stimulus intensity up to 70% MSO because of the discomfort the participants felt during stimulation. Therefore, it remains unknown whether TVMS has an upper boundary to evoke walking-like leg movements. A previous study in cats reported that bilateral hindlimb locomotion disappears when the intensity of spinal dorsal root electrical stimulation is too high (Barthélemy et al., 2007). In our study, the out-of-phase leg coordination pattern was preserved at up to 70% MSO (Figure 3B), indicating that the tolerable intensity was still within the optimal intensity range for TVMS to evoke bilateral walking-like leg movements. However, at a stimulus intensity higher than that required to evoke walking-like movements (approximately ≥ 60% MSO), co-activation of intralimb flexor-extensor muscles was observed frequently (e.g., Figure 6, left gluteus maximus and left iliopsoas). We assume that this was due to the activation of the high-threshold ventral nerve roots, in which the direct activation of motor axons induces muscle contractions. To support this assumption, a relatively uniform size of EMG amplitude was observed during the period of TVMS trains in the muscle acting as an antagonist for the observed hip joint movement (i.e., left gluteus maximus, Figure 5). Regardless of that, the stride length of the evoked walking-like movements kept increasing up to the highest intensity tested (Figure 5B), suggesting that co-activation of the intralimb flexor-extensor muscles elicited by TVMS at an intensity no higher than 70% MSO would have a minor effect on the generation of walking-like movements resulting from the activation of the spinal locomotor circuitry in the human lumbar spinal cord.

Similar to studies using transcranial magnetic stimulation, the current study determined a stimulus hot-spot prior to the testing by determining the site where the most pronounced bilateral alternative leg movement was evoked by a fixed TVMS intensity (see section “Materials and methods”). Although it could be assumed that the stimulus susceptibility for evoking the walking-like movement simply declines at increasing distance from the hot-spot (Thickbroom et al., 1998), we did not systematically investigate the effect of stimulus location on the pattern of evoked leg movement. To explore mechanisms underlying the transition of the evoked movement pattern further, we intend to investigate the topographic dependence of TVMS in future research.

The current study certainly demonstrated that rhythmically controlled TVMS was feasible to evoke the bilateral rhythmic leg muscles activity that engaged in the hopping-like and the walking like movement. However, we cannot completely exclude another possibility that non-neurophysiological factors also contributed to the rhythmic leg movements. Viscoelasticity of the body parts and/or pendulum action of the apparatus might be complicit in maintaining the cycle of rhythmic leg movements.

### Clinical implications

Epidural spinal cord stimulation has been used increasingly in gait rehabilitation for neurological disorders such as SCI (Angeli et al., 2014, 2018; Gill et al., 2018; Wagner et al., 2018; Zaaya et al., 2021). For individuals with severe SCI, electrical stimulation with chronically implanted epidural electrodes has been successful in restoring lost locomotor function (Angeli et al., 2018; Gill et al., 2018; Rowald et al., 2022). Compared with epidural stimulation, percutaneous spinal stimulation with electric or magnetic pulses has a lower risk of adverse events (Laskin et al., 2022), which is supported by our recent work showing that the use of repetitive TVMS under closed-loop control induces no serious adverse events in individuals with chronic SCI and uninjured healthy adults (Sasada et al., 2021). Additionally, magnetic stimulation generates less discomfort compared with percutaneous electrical stimulation due to lower current density in the skin, allowing for high frequency stimulation with suprathreshold intensity for leg muscles. These advantages suggest that TVMS will increase the options for gait rehabilitation using spinal stimulation. Our findings indicate that TVMS requires an optimal intensity of approximately ≥40% MSO to evoke bilateral rhythmic walking-like leg movements. On the basis of our previous work (Sasada et al., 2021) and verbal feedback from the current study participants, this intensity did not cause an unpleasant sensation, suggesting that TVMS can be a good alternative approach to restore impaired gait function and encourage recovery in individuals in whom epidural stimulation is not available. We intend to investigate the input-output properties of TVMS and its neurophysiological impacts on the spinal locomotor circuitry in individuals with SCI to develop a new rehabilitation therapy.

## Data availability statement

The original contributions presented in this study are included in the article/Supplementary material, further inquiries can be directed to the corresponding author.

## Ethics statement

The studies involving human participants were reviewed and approved by the Local Ethics Committee of the Tokyo Metropolitan Institute of Medical Science. The patients/participants provided their written informed consent to participate in this study.

## Author contributions

KKw, TT, and YN conceived and designed the experiments. KKw and TT performed the experiments, analyzed the data, and prepared the figures. All authors wrote the manuscript and approved the final version of the manuscript submitted for publication.
